# Evolutionary innovations driving abiotic stress tolerance in C_4_ grasses and cereals

**DOI:** 10.1093/plcell/koab205

**Published:** 2021-08-13

**Authors:** Jeremy Pardo, Robert VanBuren

**Affiliations:** 1 Department of Plant Biology, Michigan State University, East Lansing, Michigan 48824, USA; 2 Department of Horticulture, Michigan State University, East Lansing, Michigan 48824, USA; 3 Plant Resilience Institute, Michigan State University, East Lansing, Michigan 48824, USA

## Abstract

Grasslands dominate the terrestrial landscape, and grasses have evolved complex and elegant strategies to overcome abiotic stresses. The C_4_ grasses are particularly stress tolerant and thrive in tropical and dry temperate ecosystems. Growing evidence suggests that the presence of C_4_ photosynthesis alone is insufficient to account for drought resilience in grasses, pointing to other adaptations as contributing to tolerance traits. The majority of grasses from the Chloridoideae subfamily are tolerant to drought, salt, and desiccation, making this subfamily a hub of resilience. Here, we discuss the evolutionary innovations that make C_4_ grasses so resilient, with a particular emphasis on grasses from the Chloridoideae (chloridoid) and Panicoideae (panicoid) subfamilies. We propose that a baseline level of resilience in chloridoid ancestors allowed them to colonize harsh habitats, and these environments drove selective pressure that enabled the repeated evolution of abiotic stress tolerance traits. Furthermore, we suggest that a lack of evolutionary access to stressful environments is partially responsible for the relatively poor stress resilience of major C_4_ crops compared to their wild relatives. We propose that chloridoid crops and the subfamily more broadly represent an untapped reservoir for improving resilience to drought and other abiotic stresses in cereals.

## Teaser

Chloridoid grasses have evolved unique adaptations to adverse environments and represent an untapped reservoir for improving resilience to drought and other abiotic stresses in cereals.

## Introduction

The earliest grasses emerged between 55 and 70 million years ago, and now dominate ecosystems covering 30%–40% of ice-free land (Linder and Ferguson, 1985; Jacobs et al., 1999; Kellogg, 2001; Blair et al., 2014). Grasslands typify environments that are too stressful to support trees. In the Arctic, grasses prevail north of the boundary where low temperatures and permafrost prevent tree growth (Scheffer et al., 2012), while in warmer regions, lower mean annual precipitation and/or frequent disturbances such as wildfires, favor open savannas over wooded ecosystems (Higgins et al., 2000; Sankaran et al., 2005). The ability of grasses to colonize these relatively harsh environments is enabled by a network of unique anatomical, physiological, and molecular adaptations that combat issues related to water, temperature, salinity, and excess light stresses (Linder et al., 2018). Much of the resilience in grasses has been attributed to the evolution of C_4_ photosynthesis (Osborne and Freckleton, 2009; Christin et al., 2013), an optimized carbon concentration mechanism that reduces photorespiration and improves water-use efficiency (WUE). Other adaptations such as low critical leaf water potential and a modified leaf anatomy also contribute to drought tolerance in grasses (Balsamo et al., 2006; Nunes et al., 2020). Most resilience traits are either conserved or widespread in the grass family. For instance, grasses share a unique stomatal structure, which is thought to be more efficient than the stomata of other plants (Stebbins and Shah, 1960; Franks and Farquhar, 2007; Chen et al., 2017; Nunes et al., 2020). Similarly, C_4_ photosynthesis is widespread in the grass family, representing ∼42% of all grass species (Osborne et al., 2014). Tolerance to abiotic stressors is evolutionarily labile in grasses, despite the prevalence of underlying traits that enable stress tolerance. Cold, salt, and desiccation tolerance are all thought to have evolved independently multiple times within grasses (Bennett et al., 2013; Gaff and Oliver, 2013; Schubert et al., 2019).

**Figure 1:**
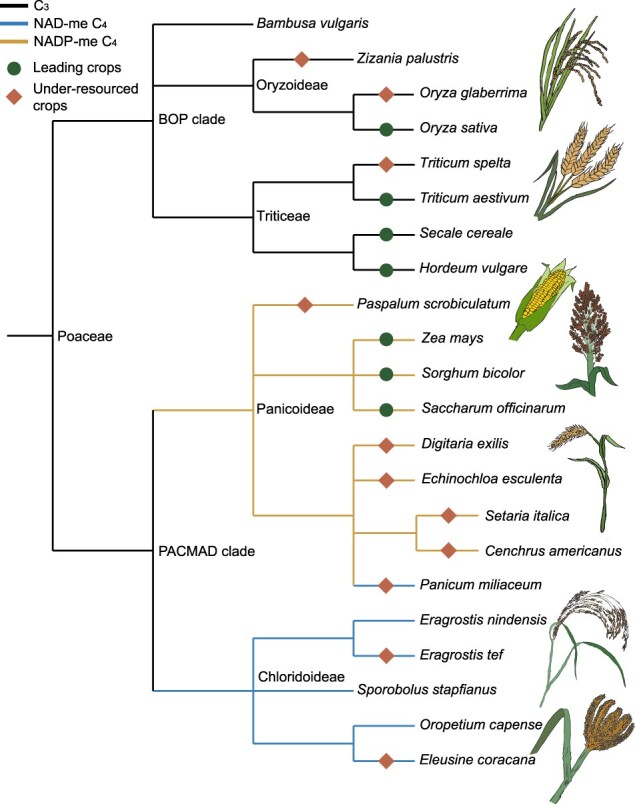
Phylogeny of agronomically important C_3_ and C_4_ grasses. The two major clades of grasses, the BOP and PACMAD are shown. Leading or underresourced crop species are highlighted by green circles and orange diamonds, respectively.

The majority of species in the grass family falls into two evolutionarily and phenotypically distinct clades, BOP (Bambusoideae, Oryzoideae, and Pooideae subfamilies) and PACMAD (Panicoideae, Arundinoideae, Chloridoideae, Micrairoideae, Aristidoideae, and Danthonioideae subfamilies), named for the subfamilies they contain (Figure 1; Grass Phylogeny Working Group II, 2012). Most species in the BOP clade are classified as cool-season grasses with distributions in temperate climates, where C_3_ outperforms C_4_ photosynthesis. Within BOP, the Bambusoideae and Oryzoideae subfamilies are generally native to warmer climates and include the agronomically important species bamboos (*Bambusa* sp.) and rice (*Oryza sativa*), respectively. Pooideae is the largest subfamily of grasses and includes the temperate cereals wheat (*Triticum aestivum*), barley (*Hordeum vulgare*), oat (*Avena sativa*), and rye (*Secale cereale*), as well as most pasture grasses. All BOP clade grasses utilize the C_3_ pathway of photosynthesis, and most independent origins of frost tolerance in grasses are found within the Pooideae (Schubert et al., 2019). Conversely, grasses in the PACMAD clade are mostly distributed in warm temperate and tropical regions. PACMAD contains all known origins of C_4_ photosynthesis in grasses, the majority of salt tolerance origins, and all but one origin of desiccation tolerance (Sage, 2004; Bennett et al., 2013; Marks et al., 2021). The agriculturally important PACMAD grasses belong to two subfamilies: sugarcane (*Saccharum officinarum*), maize (*Zea mays*), sorghum (*Sorghum bicolor*), and various millets are in Panicoideae and the under-resourced grain crops finger millet (*Eleusine coracana*) and teff (*Eragrostis tef*) are in the Chloridoideae. In this review, we focus on the PACMAD grasses and the evolution of the abiotic stress tolerance that empowered their dominance and diversification. We ask which factors fostered the evolution of stress tolerance in these grasses, and why all C_4_ PACMAD grasses are not drought-tolerant.

The Panicoideae and Chloridoideae have very different evolutionary histories that have shaped their respective aridity tolerance. In general, panicoid grasses are taller, more ecologically dominant, and less stress-tolerant than the shorter, more stress-resilient chloridoid grasses (Liu et al., 2012; Liu and Osborne, 2015). Panicoid grasses are also better represented among major crops, and of nine C_4_ grasses in the United Nations Food and Agriculture Organization global crop production database, only two, finger millet and teff, are chloridoid grasses (Sage and Zhu, 2011). The underrepresentation of chloridoid grasses among worldwide crops may be explained by the hypothesis of a tradeoff between yield and stress tolerance (Silva et al., 2019). If true, perhaps chloridoid grasses have underlying traits that simultaneously confer drought tolerance but also limit production, making them less suitable crop plants. Alternatively, the low number of domesticated chloridoid grasses may stem from their ecological underrepresentation in the main centers of origin for crop plants. Panicoid species are dominant in mesic environments that were most conducive to the development of agriculture, compared to the more arid regions where chloridoid grasses dominate (Liu and Osborne, 2015). If this is the case, chloridoid species may represent an untapped resource for developing the next generation of climate-resilient crops. Here, we highlight the resilience traits distinguishing panicoid and chloridoid grasses and discuss the potential of using chloridoid species to improve the climate resilience of agriculture.

## What makes PACMAD grasses so resilient?

Grasses have evolved unique anatomical, physiological, molecular, and life history traits to thrive in poor or dynamic environments. Evolutionary innovations underlying resilience, such as modified stomata, C_4_ photosynthesis with Kranz anatomy, salt glands, and desiccation tolerance, arose independently in grasses (Sage, 2004; Grass Phylogeny Working Group II, 2012; Bennett et al., 2013; Gaff and Oliver, 2013). Other adaptations such as high WUE, improved leaf water potential under drought conditions, and deep fibrous root systems in grasses represent stepwise improvements on conserved mechanisms found in all plants. Some of these traits are conserved widely across grasses, but many are uniquely or more frequently found in the PACMAD clade. The C_4_ members of the PACMAD clade are especially drought-resilient compared to C_3_ members of the clade (Pau et al., 2013; Taylor et al., 2014).

Grasses with C_4_ photosynthesis cover ∼18% of vegetated land area, especially in tropical, arid, and semi-arid regions (Still et al., 2003). C_4_ grasses are also crucial for agriculture, with two C_4_ species (maize and sugarcane) leading all other plants in terms of global production. Water availability is thought to have been a major driving force of C_4_ grass evolution and diversification (Osborne and Sack, 2012). However, not all C_4_ grasses are from arid environments, and tolerance to drought varies widely across C_4_ grasses. For example, the so-called resurrection grasses, such as lovegrass (*Eragrostis nindensis*) and *Oropetium capense*, are able to equilibrate to low atmospheric moisture for months without dying, while other C_4_ species such as switchgrass (*Panicum hemitomon*) are semi-aquatic, requiring regular flooding to survive (Gaff, 1971; Kirkman and Sharitz, 1993). This raises the question: what factors enable drought tolerance in the C_4_ PACMAD grasses, if C_4_ photosynthesis per se is not the sole driver of stress tolerance?

**Figure 2:**
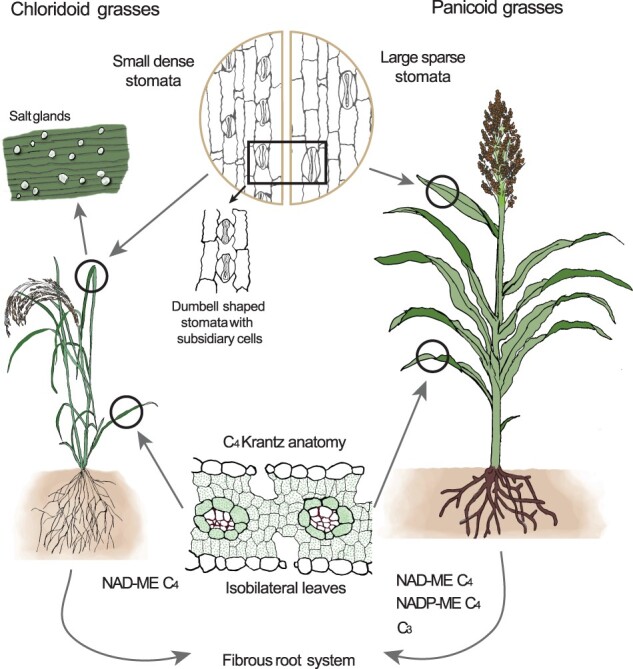
Evolutionary innovations contributing to stress tolerance in C_4_ grasses. Several shared and unique adaptations in chloridoid grasses (left) and panicoid grasses (right) are shown.

The 19th century architect Louis Sullivan famously stated that “form ever follows function” (Tubbs, 2015). This saying has long been applied to biology to describe how structure and function are related. This principle applies particularly well to abiotic stress adaptation among PACMAD grasses, where their anatomy is intimately linked to their resilience. Stomatal anatomy is one example of an anatomical trait conferring resilience across all grasses. Grasses have a unique stomatal structure with elongated dumbbell-shaped guard cells and two subsidiary cells (Stebbins and Shah, 1960; Figure 2). This morphology allows faster stomatal responses than those of the kidney-shaped guard cells of eudicots and most nongrass monocots, resulting in higher WUE (McAusland et al., 2016; Lawson and Vialet-Chabrand, 2019). In addition to the structure of guard cells, the arrangement and density of stomatal pores is another important factor in determining drought tolerance. The majority of grasses have either hypostomatic leaves, where the stomatal pores are primarily on the abaxial leaf surface, or amphistomatic leaves, where the pores are roughly equally distributed between the adaxial and abaxial surfaces. Amphistomatic leaves allow for more efficient CO_2_ diffusion into the leaf and therefore greater maximum photosynthetic rates (Hardy et al., 1995). In eudicots with dorsoventral leaf anatomy, leaves are often held perpendicular to the axis of irradiance and amphistomaty comes at the cost of greater evapotranspiration. However, grasses have isobilateral leaves that are often held parallel to the axis of irradiance. The deeper placement of veins in isobilateral leaves and the more vertical leaf angle of these grasses overcome the WUE cost of amphistomatic leaves (Drake et al., 2019). Among grasses, amphistomatic leaves are more prevalent among C_4_ species, particularly among those adapted to arid environments with high irradiance (Mott et al., 1982; Drake et al., 2019).

## Contribution of the “C_4_ syndrome” to water deficit stress tolerance

C_4_ photosynthesis is a central trait that has enabled PACMAD grasses to survive arid environments. At its heart, C_4_ photosynthesis is a carbon concentrating mechanism; however, it is not exclusively a biochemical trait, as a modified leaf anatomy is needed for C_4_ to operate efficiently. Thus, C_4_ photosynthesis has been labeled as a “syndrome” of both anatomical and biochemical traits (Laetsch, 1974). In 1884, the botanist Gotleib Haberlandt described the “Kranz” anatomy of certain plants, whereby a ring of large bundle sheath cells around the vascular bundles containing many chloroplasts is surrounded by a second, sparser, ring of smaller mesophyll cells (Figure 2) (Haberlandt, 1884; Lundgren et al., 2014). This anatomy was later associated with C_4_ photosynthesis, and the majority of C_4_ species, including grasses, has the Kranz-type leaf anatomy (El‐Sharkawy and Hesketh, 1965; Edwards et al., 2004; Lundgren et al., 2014). While primarily thought of as supporting C_4_ biochemistry, Kranz anatomy also influences WUE and drought tolerance. Indeed, the large bundle sheath cells increase the hydraulic capacitance of the leaf, which may help buffer against the sudden increases in evapotranspiration that are common in open environments (Sage, 2001). C_4_ species with Kranz type anatomy have shorter interveinal distances than in C_3_ species (Sage, 2001; Griffiths et al., 2013), and an increased vein density facilitates C_4_ biochemistry by minimizing the diffusion distance between mesophyll and bundle sheath cells. Shorter interveinal distances also result in higher leaf hydraulic conductance. In C_3_ species, leaf hydraulic conductance is positively correlated with maximum CO_2_ assimilation rates (Brodribb et al., 2007), which is thought to result from higher stomatal and mesophyll conductance of CO_2_ in plants with higher leaf hydraulic conductance. However, under dry conditions (high vapor pressure deficit), increased leaf hydraulic conductance results in lower WUE (Sinclair et al., 2008; Rzigui et al., 2018). Thus, C_3_ species experience a tradeoff between carbon gain and WUE. However, in C_4_ species, net assimilation is decoupled from hydraulic conductance (Ocheltree et al., 2016). C_4_ biochemistry can tolerate reduced stomatal conductance, which conserves water. Furthermore, C_4_ species adapted to dryer environments have greater mesophyll conductance and lower hydraulic conductance as compared to C_4_ species from wet environments (Pathare et al., 2020). Therefore, these species can increase hydraulic safety while maintaining high CO_2_ assimilation rates.

The anatomical traits that enable C_4_ biochemistry are thought to predate the evolution of the biochemical carbon concentrating mechanism, which has evolved at least 22 times independently within the grass family (Grass Phylogeny Working Group II, 2012). In the C_3_ ancestors of these modern C_4_ lineages, higher hydraulic conductance likely came at the cost of WUE. However, after the carbon concentrating mechanism arose, there has consistently been selection for lower leaf hydraulic conductance while maintaining maximum CO_2_ assimilation rate within C_4_ grasses (Zhou et al., 2020). Thus, lineages where C_4_ arose earlier, and those with faster evolutionary rates, tend to have lower leaf hydraulic conductance and higher WUE (Zhou et al., 2020). The Chloridoideae subfamily likely contains the oldest origin of C_4_ photosynthesis and, unlike the Panicoideae subfamily, the ancestor of core chloridoid grasses was likely C_4_ (Christin et al., 2008). Thus, chloridoid lineages have had the greatest amount of time since the introduction of C_4_ biochemistry, and thus the most time to respond to the strong selective pressure favoring reduced hydraulic conductance. Consistent with this phylogenetic history, modern chloridoid grasses generally have lower leaf hydraulic conductance than panicoid grasses (Liu and Osborne, 2015).

## Relation between the C_4_ biochemical pathway and WUE

The C_4_ pathway optimizes WUE, and C_4_ grasses tend to occupy drier and more exposed habitats than their C_3_ relatives (Edwards and Still, 2008). In C_4_ photosynthesis, phosphoenolpyruvate carboxylase (PEPcase) catalyzes the reaction fixing inorganic bicarbonate (HCO_3_), which is in equilibrium with CO_2_, into organic acids. These organic acids are then transported to bundle sheath cells where they are decarboxylated, raising the bundle sheath CO_2_ concentration and allowing Rubisco to operate more efficiently (Kellogg, 2013). The higher affinity of PEPcase for its substrate HCO_3_ compared to that of Rubsico for CO_2_, along with the C_4_ carbon concentrating mechanism more generally, allows C_4_ plants to operate at lower mesophyll CO_2_ concentrations than C_3_ plants. Consequently, they are able to maintain lower stomatal conductance, resulting in higher instantaneous WUE (Ghannoum et al., 2011). Under drought stress, leaf-level WUE often increases, as water savings from stomatal closure are greater than the reduction in CO_2_ assimilation due to inhibition of photosynthesis.

C_4_ grasses are classified into three distinct subtypes based on their biochemistry: NADP-dependent malic enzyme (NADP-me), NAD-dependent malic enzyme (NAD-me), and phosphoenolpyruvate carboxykinase (PCK). In NADP-me plants, malate is the primary C_4_ acid being transported between mesophyll and bundle sheath cells, while aspartate is the primary transported acid in NAD-me and PCK C_4_ grasses. WUE is correlated with the C_4_ subtype, with NAD-me grasses having higher WUE under drought stress than NADP-me grasses (Ghannoum et al., 2002). NAD-me C_4_ grasses are more abundant in arid regions, while NADP-me species tend to inhabit more mesic environments (Taub, 2000; Liu and Osborne, 2015). There is only a weak correlation between the distribution of PCK-based C_4_ grasses and precipitation gradients (Ghannoum, 2009). The PCK pathway is thought to be an addition to both the NAD-me and NADP-me pathways and is found at relatively equal frequency across both panicoid and chloridoid grasses. In contrast, the distribution of NADP-me and NAD-me C_4_ pathways mostly follows phylogenetic lineages. All species within the Chloridoideae subfamily are either of the NAD-me or PCK subtypes. The NAD-me subtype is thought to be the ancestral state of chloridoid grasses, from which PCK grasses arose. Panicoid grasses are mostly NADP-me with a minority of NAD-me and PCK species. These factors make it difficult to separate the influence of phylogeny and selective pressures on their biochemistry.

## Distinguishing features underlying stress tolerance in chloridoid grasses

Many of the most stress-tolerant grasses belong to the PACMAD clade, but this resilience is not uniform, and substantial variation exists between and within these clades. The Chloridoideae subfamily is arguably the most stress tolerant subfamily of PACMADs, and dominates in arid and resource-poor subtropical and tropical deserts that are inhospitable to most grasses (Clayton and Renvoize, 1986). The degree of tolerance in chloridoid grasses is linked to their C_4_ subtype, as taxa with the NAD-me subtype thrive in hot and dry climates whereas PCK taxa are more common in mesic habitats. NAD-me chloridoids have an additional column of cells between the vascular bundles that is missing from PCK species and promotes leaf rolling, thus limiting transpirational water loss (Peterson et al., 2007). This anatomical adaptation facilitates tighter leaf rolling in NAD-me chloridoid grasses compared to other C_4_ grasses (Liu and Osborne, 2015). The majority of chloridoid species are classified as the NAD-me photosynthetic subtype as are some of the most resilient panicoid grasses, raising the question as to whether biochemical subtype or phylogeny is a more important predictor of resilience. Habitat aridity is correlated with subfamily in C_4_ grasses, with chloridoid species occupying drier niches. However, other factors such as a preference for open habitats and shorter stature in chloridoid compared to panicoid species are also correlated with phylogeny and contribute to the overall habitat preference of chloridoid grasses for dry open environments (Liu et al., 2012). At the leaf level, anatomical traits are also strongly correlated with phylogeny; chloridoid species have higher specific leaf area, higher stomatal density, and smaller stomata than panicoid species. However, physiological traits such as leaf water potential under ambient and saturating conditions are more strongly influenced by photosynthetic subtype than phylogeny (Liu and Osborne, 2015).

It is difficult to separate the effects of phylogeny and photosynthetic subtype on habitat preference, as the two are intertwined. For example, there is a significant interaction between phylogeny and the photosynthetic subtype for certain leaf hydraulic traits (Liu and Osborne, 2015). Turgor loss point is the leaf water potential at which wilting occurs and is a function of both leaf osmotic potential and tissue flexibility (Cosgrove, 1988; Bartlett et al., 2012). Surprisingly, NAD-me species in both the chloridoid and panicoid lineages have less negative turgor loss points than either PCK or NADP-me species. However, PCK chloridoid species have more negative turgor loss points than panicoid PCK species (Liu and Osborne, 2015). A more negative osmotic potential under saturating conditions is correlated with greater osmotic adjustment under drought stress, allowing plants to maintain turgor at lower leaf water potentials. Leaf flexibility, as measured by bulk modulus of elasticity (ε), is the ratio of change in cell turgor divided by the change in relative cell volume (Steudle and Zimmermann, 1977; Touchette et al., 2014). A higher *ε* value indicates more rigid cells and theoretically would result in a more negative turgor loss point. However, in a meta-analysis of 372 species, osmotic potential at saturation was shown to be the primary driver of turgor loss point, not bulk modulus of elasticity (Bartlett et al., 2012). Plants with more flexible cells (lower ε) are able to maintain lower relative water content at the turgor loss point and contribute to a greater capacity to maintain leaf integrity under adverse osmotic conditions (Bartlett et al., 2012; Liu and Osborne, 2015). Likewise, chloridoid PCK species were shown to have more negative saturated osmotic potential and higher ε, while chloridoid NAD-me species had less negative osmotic potential and lower ε (Liu and Osborne, 2015), possibly a result of different drought response strategies, with PCK chloridoid species exhibiting tolerance through osmotic adjustment while NAD-me species employ an avoidance strategy through a higher capacity to buffer against adverse osmotic conditions (Liu and Osborne, 2015). Given that NAD-me chloridoid species tend to occur in drier habitats than PCK species, it is unexpected that they would also be less able to tolerate drought stress at a physiologically relevant level and instead employ strategies to avoid water stress. One explanation is that the prevalence of NAD-me chloridoids in dry habitats is driven not by their inherent stress tolerance, but by another feature that afforded chloridoids the ecological opportunity to radiate into dry environments. Ancestral state reconstruction indicated that the C_3_ ancestor of the Chloridoideae subfamily likely lived in dry areas (Osborne and Freckleton, 2009). The paleontologist Gaylord Simpson originally proposed the idea that “evolutionary access to ecological opportunity” may drive adaptive radiation (Simpson, 1953; Edwards and Donoghue, 2013; Stroud and Losos, 2016). In the case of the NAD-me chloridioids, perhaps features such as their preference for high-irradiance, open environments, gave these early chloridoid species evolutionary access to dry environments. Subsequent adaptations to their primarily arid environment then led to the resilience observed in this group today.

The resilience of the chloridoid subfamily is not limited to ordinary drought tolerance. Chloridoid grasses are also well represented among halophytes and desiccation-tolerant species. Drought and high salinity often co-occur, and both can cause osmotic stress in plants. Thus, cross-tolerance is common. Salinity tolerance is widely distributed across the grass phylogeny and is thought to have arisen independently over 70 times (Bennett et al., 2013). Most origins of salinity tolerance in the grass family are relatively recent, resulting in numerous small clades of halophyte grasses. However, the Chloridoideae subfamily is the exception, and likely contains ancient origins of salt tolerance (Bennett et al., 2013). Is it possible that drought tolerance in the Chloridoideae evolved through a common mechanism with salt tolerance or that one trait enabled the evolution of the other? Salinity tolerance is more prevalent among C_4_ lineages as compared to C_3_ lineages within the PACMAD clade (Bromham and Bennett, 2014). The correlation between C_4_ and salinity tolerance within PACMAD grasses has both physiological and evolutionary explanations. Salt tolerance is conferred through both ion exclusion and osmotic adjustment. Grasses with the C_4_ pathway are in general more efficient in their water use than their C_3_ counterparts, translating into the uptake of fewer ions per fixed carbon. However, many chloridoid grasses adapted to saline environments take up sodium ions but then excrete them through specialized salt glands. Salt glands are seemingly unrelated to water-deficit stress caused by drought, while osmotic adjustment is an important response to water-deficit. While all chloridoid species accumulate compatible solutes when grown in saline conditions, the primary salt tolerance mechanism is thought to be excretion through bicellular salt glands (Marcum and Murdoch, 1994; Marcum, 1999). Thus, cross tolerance alone is likely insufficient to explain the prevalence of both drought and salt tolerance within the chloridoid subfamily. Alternatively, salt tolerance, the C_4_ pathway, and drought tolerance may be correlated traits because dry environments and saline environments often co-occur. Therefore, species living in these environments face selective pressures that make all three traits adaptive (Bennett et al., 2013). Consequently, chloridoid grasses may have evolved these traits because they had the evolutionary access to overcome a selective pressure.

The idea that evolutionary access drives the prevalence of stress tolerance traits in Chloridoideae may explain the likely multiple independent origins of desiccation tolerance in this subfamily (Gaff and Oliver, 2013; Pardo et al., 2020). Desiccation tolerance is the ability of vegetative tissue to survive drying, often defined as equilibration with 50% relative humidity air or drying to 10% absolute water content, without dying (Bewley, 1979; Alpert, 2005). Vegetative desiccation tolerance relies on a combination of anatomical, biochemical, and molecular adaptations (Vander Willigen et al., 2001; Costa et al., 2017; VanBuren et al., 2017). Studies examining gene expression of vegetative tissues in desiccation-tolerant species repeatedly find high expression of genes normally expressed during seed maturation and dehydration (Mitra et al., 2013; Costa et al., 2017; VanBuren et al., 2017). It is often hypothesized that the repurposing of seed desiccation pathways for vegetative tissues drove the evolution of desiccation tolerance (Oliver et al., 2000; VanBuren, 2017). However, the transcriptional network responsible for coordinating the seed dehydration response is not activated in the leaves of the desiccation-tolerant monocot *Xerophyta humilis* (Lyall et al., 2019). Furthermore, we previously found that across five grass species, more components of the seed dehydration pathway are expressed in leaves of all species under severe drought stress, irrespective of their desiccation tolerance or susceptibility (Pardo et al., 2020). The overlap between desiccation-sensitive and -tolerant species suggests that underlying conserved drought responses allowed the subsequent evolution of desiccation tolerance. Vegetative desiccation tolerance is an uncommon trait among grasses, with only nine genera within Poaceae containing desiccation-tolerant species (Gaff and Oliver, 2013). However, most of these desiccation-tolerant genera (seven) are found within the Chloridoideae subfamily (Marks et al., 2021). This clumped distribution of desiccation tolerance across the grass family may indicate the predisposition of chloridoid grasses to evolve this trait. However, the superior drought tolerance of chloridoid grasses may have also enabled the evolution of desiccation tolerance in this group by allowing chloridoid ancestors to grow in environments where desiccation tolerance is adaptive. The ancestors of desiccation-tolerant chloridoid grasses had the evolutionary access to the selective pressure that made vegetative desiccation tolerance an adaptive trait. Desiccation-tolerant species from only distantly related lineages often co-occur in rocky, dry areas and are even the dominant flora in these specialized habitats (Conceição et al., 2007; Alcantara et al., 2015). In addition to a lack of moisture, these rocky dry areas are also open, exposing plants to high irradiance. This is perhaps also key to the evolution of desiccation tolerance, as photoprotective mechanisms are thought to play a major role in desiccation tolerance (Huang et al., 2012; Verhoeven et al., 2018; VanBuren et al., 2019). Given that desiccation-tolerant grasses are rare outside these conducive environments, it is likely that the trait is only adaptive under a particular set of environmental conditions. Thus, at a minimum, access to those environments is likely necessary, if not sufficient, to afford the opportunity to evolve desiccation tolerance. Chloridoid grasses radiated in open, high-light, dry environments, and high light is an important component of their ecological niche (Osborne and Freckleton, 2009; Liu et al., 2012). Other lineages of desiccation-sensitive PACMAD grasses cohabitate regions with tolerant Chloridoid species, but they may lack the prerequisite traits to evolve desiccation tolerance. Adaptation to high light and arid environments possibly drove the evolution of these enabling traits, which then allowed for the subsequent repeated evolution of desiccation tolerance in Chloridoideae. More broadly, once a species is established in a particular environment, it is subjected to selective pressures, which then drive adaptations to the conditions prevalent in that environment. C_4_ grasses, and particularly the Chloridoideae subfamily, diversified in dry, open, and sometimes salty environments. They therefore evolved traits to cope with these pressures, resulting in a reservoir of resilience within this group of grasses.

## Is resilience a roadblock for domestication in grasses, or a source of untapped genetic potential?

Water deficit is the greatest abiotic threat to global food production. A single drought event reduces the gross agricultural production of a nation by an average of 0.8%, according to global data collected between 1983 and 2009 (Kim et al., 2019). The prevalence and severity of drought events are forecasted to increase in many agricultural areas over the next century, and drought-associated losses will be amplified under the changing climate (Dai, 2011). The evolution and diversification of C_4_ lineages was driven largely by exposure to arid environments, and C_4_ cereals can thrive in hot, dry conditions that are too extreme for other cereals and staple crops (Osborne and Sack, 2012). Thus, C_4_ cereals should be a more central component of a stable and resilient food system under a changing climate.

The yield per hectare of C_4_ cereals and biomass grasses far exceeds that of most other crops, yet despite their relative efficiency and resiliency, this level of productivity still requires a substantial amount of water. C_4_ staples of the global food system such as maize and sugarcane are among the most water-intensive crops. High-yielding commercial maize hybrids require ∼500–750 mm of precipitation over the course of the growing season, with a peak water use of ∼7.5 mm per day (Kranz et al., 2008). To meet their water requirements, dryland maize requires a minimum of ∼600 mm of precipitation over the growing season. Sugarcane requires 1,200–2,700 mm of water over its 11- to 18-month growing season, with a peak daily water use of ∼6 mm per day. The extensive water requirements of sugarcane limit its production to areas with greater than 1,000–1,200 mm of annual precipitation (Yates and Taylor, 1986). WUE for staple C_4_ crops such as maize is high despite their high absolute water requirements. However, the maximum WUE requires substantial water input and WUE drops substantially in environments with less water (Fang et al., 2017). High precipitation or irrigation requirements are not universal across C_4_ grasses, and other less widely grown C_4_ cereals such as the chloridoids teff and finger millet and the panicoids proso millet (*Panicum miliaceum*) and fonio millet (*Digitaria* *exilis*) use far less water (Table 1). Teff is grown primarily in the arid highlands and lowlands of Ethiopia and Eritrea and requires only ∼300 mm of water during the growing season. Similarly, proso millet is regarded as having the lowest water requirement of any grain crop, using just 200–300 mm. Collectively, multiple grain species categorized as millets constitute an important global crop; however, the total production of all millet species is still far short of that from maize (Habiyaremye et al., 2016). Given the limitation that drought imposes on agricultural yields, it is surprising that less water stress-tolerant crop species dominate in terms of acreage planted. One possible explanation is a tradeoff between stress tolerance and growth. Such a tradeoff has been hypothesized to account for the generally slow growth of desiccation-tolerant species (Alpert, 2005, 2006). If a tradeoff between yield and stress tolerance exists, perhaps the most productive C_4_ cereal crops are inevitably less stress-tolerant than lower yielding but more resilient C_4_ species.

**Table 1:** Comparison of C_4_ crop water use and yield. Global average and Least Developed Countries yield data (Tonnes per Hectare) are adapted from the FAOstat database for 2019 crop yields (Food and Agriculture Organization of the United Nations, 2019). The minimum and maximum yield range (Tonnes per Hectare), growing season water requirements (mm), as well as the growing season length for each crop are adapted from the Useful Tropical Plants Database (Fern and Fern, 2014)

Crop	Scientific Name	Water Requirement (mm)	Growing Season Length (months)	Global Average Yield (T·H−1)	Least Developed Countries Yield (T·H−1)	Minimum Yield Range (T·H−1)	Maximum Yield Range (T·H−1)
Maize	*Zea mays*	500–750	4–5	5.8	1.95	1	20
Sugarcane	*Saccharum officinarum*	1,200–2,700	11–18	72.8	57.74	50	150
Sorghum	*Sorghum bicolor*	450–650	3–4	1.45	0.89	2	6
Teff	*Eragrostis tef*	300	2–5	0.89^a^	0.67^a^	0.2	4.5
Finger millet	*Eleusine coracana*	350	3–6	0.89^a^	0.67^a^	0.25	5
Proso millet	*Panicum miliaceum*	200–300	2–3	0.89^a^	0.67^a^	0.45	2
Pearl millet	*Cenchrus americanus*	350	2–3	0.89^a^	0.67^a^	0.25	8
Fonio	*Digitaria exilis*	250–350	2–3	0.76	0.81	0.6	1

^a^ All millets including pearl millet, proso millet, finger millet, and teff are grouped together in the FAOSTAT Database.

Drought resilience does not always negatively correlate with yield. For example, yield comparisons of the C_4_ panicoid crops maize, sorghum, and pearl millet (*Cenchrus americanus*) in a semi-arid environment revealed that maize is the highest yielding crop, followed by sorghum, with pearl millet having the lowest yield (Muchow, 1989), despite the fact that sorghum and pearl millet are more drought-resistant than maize. However, in drier environments where maize yields dropped below 6.4 metric tons per hectare, sorghum was more productive (Staggenborg et al., 2008). Cross-species comparisons are suggestive of a tradeoff between yield and stress tolerance, whereby the higher yielding species outperform the more stress-tolerant cereals in all but the most stressful environments. However, within-species analysis suggests otherwise. An examination of the commercial “drought-tolerant” maize hybrids from three major seed companies found that the drought-tolerant lines outperformed drought-susceptible cultivars in dry environments with no yield penalty under adequate moisture (Adee et al., 2016). Similarly, adoption of drought-tolerant maize lines developed by the International Maize and Wheat Improvement Center (CIMMYT) increased maize yield in Uganda by 15% (Simtowe et al., 2019). In sorghum, the stay-green phenotype, which confers resistance to senescence under terminal drought, is associated with increased yield under dry conditions but has a minimal to no yield penalty under adequate moisture (Sabadin et al., 2012).

**Figure 3:**
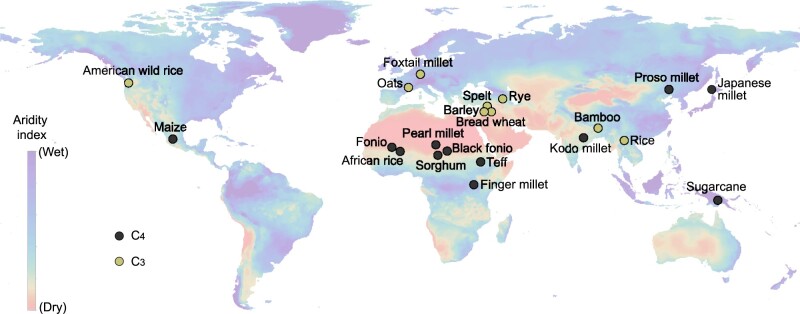
Domestication and origin of major C_3_ and C_4_ crops and cereals. The putative centers of origin for major domesticated grasses are shown with C_4_ species highlighted in black and C_3_ species highlighted in yellow. The aridity index is overlaid; blue regions are the least arid and orange regions the most arid. Data for the crop origins were adapted from (Milla, 2020).

If there is not necessarily a tradeoff between yield and drought stress resilience, what other factors might explain the relative lack of water-stress tolerance among C_4_ global staples? In the case of maize and sorghum, their respective domestication histories may explain their differences in drought resilience. Maize was domesticated in the central Balsas valley in what is now southwest Mexico (Figure 3). Today, this region receives ∼1,200 mm of rainfall annually, 80% of which falls during the wet season from June to October (Piperno et al., 2007). By contrast, sorghum was domesticated in the Kassala region of Sudan (Fuller and Stevens, 2018). This region receives only 100–400 mm of precipitation annually. Thus, the wild progenitor of sorghum was selected in a much drier environment than the maize progenitor. While the origin of divergent drought tolerance levels in maize and sorghum may be ancient, the differences in yield between the two species are actually rather recent developments. In the USA, for example, maize and sorghum yields were very similar until 1960 (Staggenborg et al., 2008). Since that time, maize yields have increased rapidly relative to those of sorghum. This yield increase might be attributed to greater funding and efforts focused on maize improvement rather than a tradeoff resulting from their diverging stress tolerance. However, even among the generally more drought-tolerant species such as sorghum and the C_4_ millet species, water availability often limits production. For example, despite its low water requirement, proso millet frequently experiences yield losses from drought due to its shallow root system (Habiyaremye et al., 2016). Alternatively, perhaps major agricultural crops are less stress-tolerant because early farmers lived in more mesic environments, and thus domesticated less stress-tolerant plants from the local flora. The emergence of agricultural societies is linked to domestication centers rich in species with abundant resources such as the Fertile Crescent in the Middle East (Figure 3; Harlan and Others, 1992; Lev-Yadun et al., 2000). In Western Africa, yam (*Dioscorea* sp.), African rice (*Oryza glaberrima*), pearl millet, and cowpea (*Vigna unguiculata*) were domesticated around the Niger River, likely in the early Holocene when the “green Sahara” slowly desertified (Hély et al., 2009; Scarcelli et al., 2019). Proso millet was domesticated in Neolithic China ∼10,000 years ago and is the earliest dry farming crop in East Asia. Proso millet was historically grown in the dryer interior regions of China, which receive 350–450 mm of water annually compared to the later domesticated Foxtail millet that dominated the wetter eastern areas of China with an average of 450–550 mm water per year (Lu et al., 2009). It is possible that C_4_ crops domesticated in drier areas experienced stronger selection for drought tolerance over yield. Consequently, the more resilient C_4_ crops were possibly not selected as intensely for yield.

The traits that shaped the domestication of millets are different from the key innovations that characterize C_3_ and C_4_ cereals. Most millets were domesticated in semi-arid regions of Africa and India, where selection favored stable and reliable yields in drought-plagued and low rainfall areas (Figure 3; Doggett, 1989). Finger millet was domesticated in the dry highlands of Ethiopia and Uganda, with a second center of diversity in the Himalayas of Nepal and India (Hilu and De Wet, 1976; Hilu et al., 1979). Teff was domesticated in similarly arid regions of the Ethiopian highlands (Costanza et al., 1979; D’Andrea, 2008). Fonio (*D.* *exilis*) and iburu (*D. iburua*) were domesticated in the central delta and Jos plateau of Nigeria, respectively, and drought-tolerant iburu is intercropped with fonio as a fail-safe under low rainfall (De Wet, 1986). Millets are often referred to as ‘orphan crops’ because their yield is considerably lower than leading cereals, and they have undergone less intensive breeding and selection than other cereal crops. This term is somewhat of a misnomer; however, as most millets underwent intensive selection during early domestication, but the target traits are not normally associated with high-yielding cereals. Millets like teff and fonio have small grains, are susceptible to shattering and lodging, and are generally low-yielding, but they produce dependable yields under arid and poor conditions that are unsuitable for other cereals (Jideani and Akingbala, 1993; Tadesse, 1993). Teff and fonio are fast-maturing, and teff is often used as a “rescue crop” for a late season harvest after another crop fails due to drought (Tefera et al., 2001). The fast maturation of millets may come as a tradeoff, as a shorter vegetative stage means less net assimilation across the growing season, and ultimately lower crop yields. Teff is still morphologically similar to its wild progenitor *Eragrostis pilosa*, with overlapping ranges in plant architecture and seed size, but larger and more numerous panicles (Ingram and Doyle, 2003). The natural stress resilience observed in *E. pilosa* has been maintained throughout domestication and selection in teff, presumably in parallel with modest gains in yield. This suggests that resilience is not a roadblock in grasses and that cereals can be selected for higher yields while maintaining stress tolerance.

Researchers and businesses have expended considerable effort to improve resilience of major crops such as maize. For example, 20% of US corn belt acres are now planted with drought-tolerant maize hybrids (McFadden et al., 2019; Messina et al., 2020). The focus on improving drought tolerance in maize has come at the expense of the production of more drought-tolerant cereals such as sorghum (Bhagavatula et al., 2013; Mundia et al., 2019). Despite the improvements in resilience of major crops, naturally resilient cereals maintain a greater degree of stress tolerance than drought-tolerant maize. These cereals can be used to reclaim semi-arid or resource-poor land that is typically not suitable for agriculture. They also represent an opportunity to improve the resilience of agriculture more generally. Chloridoid grasses dominate in stressful environments, providing them evolutionary access to the ecological conditions necessary to evolve stress adaptations. The crop plants derived from this group share many of those adaptations, providing a strong base of resilience within these species. Conventional breeding is constrained by the available pool of genetic variation for drought tolerance traits within a given species. Efforts to improve the resilience of major crops such as maize are consequently also constrained by genetic variation, and biotech-based approaches are needed to exceed the natural tolerance found within existing germplasm. Conversely, a renewed focus on improvement of agronomic traits in naturally stress-tolerant cereals may lead to the development of crops that are simultaneously productive and resilient. Thus, we propose that more research focus is warranted on stress-tolerant cereals generally and the chloridoid subfamily in particular.

## Funding

This work was supported by NSF Grant MCB‐1817347 (to R.V.) and by predoctoral training award T32-GM110523 from the National Institute of General Medical Sciences of the NIH (to J.P.).

*Conflict of interest statement*. None declared.

## Notes

Senior author.

J.P. and R.V. designed and conceived the review topics and wrote the paper.

The author responsible for distribution of materials integral to the findings presented in this article in accordance with the policy described in the Instructions for Authors (https://academic.oup.com/plcell) is: Robert VanBuren (bobvanburen@gmail.com).
